# Metabolic regulation of pyroptotic cell death expands the therapeutic landscape for treating inflammatory disease

**DOI:** 10.1038/s41392-021-00467-w

**Published:** 2021-01-29

**Authors:** David E. Place, Thirumala-Devi Kanneganti

**Affiliations:** Department of Immunology, St. Jude Children’s Research Hospital, Memphis, TN USA

**Keywords:** Immunology, Inflammation, Drug discovery

A recent study in *Science* by Fiachra Humphries et al.^1^ reveals an important role for the metabolic intermediate fumarate in reducing gasdermin D-mediated pyroptosis. Their work adds a new inhibitor to the growing toolkit with the potential for treating pyroptosis-driven inflammatory diseases.

Pyroptosis is an inflammatory programmed cell death pathway initiated by direct sensing of pathogen-derived ligands or indirect sensing of changes to the normal cell state through sensors that can activate the inflammasome. Caspase-1 is recruited to the multimeric inflammasome to become activated and direct downstream pyroptosis and maturation of the proinflammatory cytokines IL-1β and IL-18. Similarly, caspase-11 directly senses cytosolic lipopolysaccharide (LPS) to drive pyroptosis. The lytic cell death associated with pyroptosis mediates clearance of pathogens and release of inflammatory cytokines. However, dysregulation of inflammasome activation is associated with many autoinflammatory diseases and cancer progression.

In 2015, researchers identified gasdermin D (GSDMD) as a key substrate of caspase-1 and caspase-11 (mouse) or the human homolog caspase-4/5.^2,3^ Cleavage of the linker domain of GSDMD releases the autoinhibitory C-terminal domain and allows the N-terminal domain to oligomerize and form pores on the plasma membrane of cells. The pore-forming activity of GSDMD during pyroptosis was found to be critical for cell death and facilitating the release of IL-1β and IL-18. The gasdermin (GSDM) family in humans includes GSDMA, GSDMB, GSDMC, GSDMD, GSDME (or DFNA5), and PJVK (or DFNB59), while mice lack GSDMB but possess GSDMD, GSDME, and GSDMA (GSDMA1-3), and GSDMC (GSDMC1-4) homologs. The N-terminal domains of GSDMA, GSDMB, GSDMC, and GSDME can also induce cell death similar in morphology to GSDMD-mediated pyroptosis, but the conditions under which these other GSDM family members are activated are still poorly understood. Recently, caspase-3-mediated cleavage of GSDME was found to promote a lytic form of cell death similar to GSDMD-mediated pyroptosis. *Gsdmd*^−^^/−^ mice are viable and developmentally normal, suggesting GSDMD is largely involved in regulating inflammatory cell death. Whether GSDM family members have a role in non-immune cells requires further study.

Recent studies have identified inhibitors of GSDMD function. In 2018, necrosulfonamide (NSA) was found to inhibit GSDMD function, likely through alkylating a key cysteine residue (Cys191 in human or Cys192 in mouse), which disrupts the ability of the N-terminal domain of GSDMD to oligomerize and form membrane pores.^4^ Treating mice with NSA protected them from LPS shock.^4^ The FDA-approved drug disulfiram was also recently found to specifically inhibit GSDMD pore formation but not caspase-1 or GSDMD cleavage by covalent addition of dithiodiethylcarbamoyl (DTC) at Cys191/192.^5^ Interestingly, this study found that NSA treatment inhibited the cleavage of caspase-1, GSDMD, and IL-1β, while disulfiram treatment only inhibited GSDMD pore formation. Disulfiram, similar to NSA, also reduced mortality in a mouse model of LPS shock. Together, these studies also established a critical role for the Cys191/192 residue in GSDMD inhibitor function.

In a recent publication by Fiachra Humphries et al.^1^ in *Science*, the citric acid cycle metabolic intermediate fumarate was also shown to reduce pyroptosis (Fig. 1). To study the effects of fumarate on pyroptosis, the authors utilized cell membrane-permeable dimethyl fumarate (DMF) or inhibited the citric acid cycle enzyme fumarase (also known as fumarate hydratase) to accumulate increased levels of fumarate. Mechanistically, fumarate modifies the key cysteine Cys191/Cys192 of GSDMD by forming *S*-(2-succinyl)-cysteine. This modification broadly prevented GSDMD-mediated cell death and cytokine release. DMF treatment also modified other GSDMD cysteine residues, suggesting succination may have other off-target effects. Treatment with exogenous DMF reduced both the cleavage of GSDMD and the interaction between GSDMD and caspase-1 while also leaving caspase-1 activation intact. Further experiments showed that succination broadly alters GSDMD protein-protein interaction and function. In addition to limiting GSDMD activity, DMF also reduced the cleavage of GSDME in GSDMD-deficient cells treated with the pyroptosis-inducing trigger LPS plus nigericin, potentially via succination of GSDME reactive cysteines. Treatment of mice with fumarate reduced mortality following LPS shock and limited disease in mouse models of familial Mediterranean fever (FMF) and experimental autoimmune encephalitis (EAE).

The expanding repertoire of molecules that inhibit pyroptosis through different mechanisms and at different steps has important implications for treating inflammatory diseases. Multiple inhibitors have been identified that target upstream inflammasome activation. Notably, NLRP3 inhibitors (e.g., MCC950; OLT1177) have been proposed to treat autoinflammatory diseases including multiple sclerosis and arthritis. Additionally, monoclonal antibodies and recombinant proteins that inhibit IL-1 signaling (anakinra, canakinumab, rilonacept) have been used extensively to treat autoinflammatory diseases and may have a role in limiting cancer. Because GSDM family members (including GSDMD and GSDME) mediate both the membrane lysis of dying cells as well as the release of inflammatory cytokines and DAMPs, there is also continued interest in identifying inhibitors that regulate GSDM pore-dependent release of inflammatory mediators (including HMGB1 and inflammasome-dependent cytokines) or alter pore-mediated changes in ion homeostasis. While current GSDMD inhibitors all functionally modify Cys191/192 to achieve their inhibitory activity, making synergy between them unlikely, differences in bioavailability/pharmacokinetics in tissues may constrain their use. The development of novel inhibitors that target the GSDM family or combining inhibitors that target discrete steps in pyroptosis signaling may prove beneficial.

One of the major limitations of inflammasome inhibitors has been off-target effects on other programmed cell death pathways and inflammatory signaling pathways. Indeed, current GSDMD inhibitors each modify reactive cysteines, which is likely to also affect caspases, which are cysteine-dependent proteases. Necrosulfonamide has also been shown to inhibit necroptosis by targeting a reactive cysteine residue of another pore-forming executioner protein, MLKL. Additionally, DMF inhibited both GSDMD and GSDME cleavage and modified multiple cysteine residues on each,^1^ which further indicates off-target effects may need to be overcome. This work also found that inhibiting the citric acid cycle enzyme fumarase could inhibit GSDMD-dependent cell death,^1^ suggesting alternative strategies for inhibiting pyroptosis. Further study into the molecular mechanisms of action for the Cys-reactive inhibitors that have been identified will clarify how they function differently from one another. While major hurdles remain in understanding the specific role each of these different inhibitors may have in treating autoinflammatory disease, research into this area is likely to have major therapeutic implications.

**Fig. 1:**
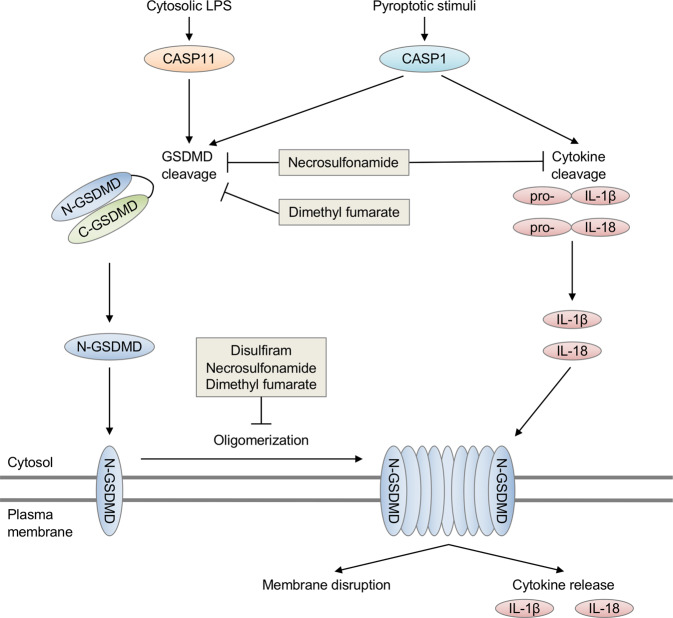
Inhibition of gasdermin D-mediated pyroptosis by necrosulfonamide, disulfiram, and dimethyl fumarate. Pyroptosis is a lytic cell death pathway activated downstream of caspase-1 and caspase-11. Activation of caspase-1/11 results in catalytic cleavage of the substrate gasdermin D (GSDMD), which releases the autoinhibitory C-terminal domain (C-GSDMD) from the pore-forming N-terminal domain (N-GSDMD). Cleavage results in N-GSDMD insertion into the plasma membrane and N-GSDMD oligomerization, forming a pore and leading to membrane disruption. Caspase-1 cleavage of IL-1β and IL-18 is also required for cytokine activation, while pore formation by GSDMD facilitates cytokine release from the cytosol. Inhibitors of GSDMD activity include necrosulfonamide (via alkylation), disulfiram (via the addition of dithiodiethylcarbamoyl [DTC]), and dimethyl fumarate (via succination), which have each been shown to covalently modify the Cys191 residue of murine GSDMD, blocking its activity. In addition to directly targeting GSDMD, off-target effects of these compounds on caspase-1 cleavage and activation have been observed

## References

[1] Humphries, F. et al. Succination inactivates gasdermin D and blocks pyroptosis. *Science*10.1126/science.abb9818 (2020). 10.1126/science.abb9818 PMC8744141 32820063

[2] Kayagaki N (2015). Caspase-11 cleaves gasdermin D for non-canonical inflammasome signalling. Nature.

[3] Shi J (2015). Cleavage of GSDMD by inflammatory caspases determines pyroptotic cell death. Nature.

[4] Rathkey JK (2018). Chemical disruption of the pyroptotic pore-forming protein gasdermin D inhibits inflammatory cell death and sepsis. Sci. Immunol..

[5] Hu JJ (2020). FDA-approved disulfiram inhibits pyroptosis by blocking gasdermin D pore formation. Nat. Immunol..

